# Single‐Cell Profiling of Pediatric High‐Grade Gliomas Reveals OPC‐Like Subpopulations Driving Tumorigenic Lineage Transitions

**DOI:** 10.1002/pdi3.70027

**Published:** 2025-09-24

**Authors:** Tian Tian, Chong Huang, Lusheng Li

**Affiliations:** ^1^ Department of Neurosurgery National Research Center for Child Health and Disorders Chongqing Key Laboratory of Translational Medical Research in Cognitive Development and Learning and Memory Disorders Children's Hospital of Chongqing Medical University Chongqing China

**Keywords:** cell lineage transition, pediatric high‐grade gliomas, tumor heterogeneity, tumor microenvironment characteristics

## Abstract

Pediatric high‐grade gliomas (pHGG) were first defined as a distinct entity in the 2021 fifth edition of the WHO classification of tumors of the central nervous system. These tumors, designated primarily as Grade 4, include the following subtypes: (1) diffuse midline glioma with H3‐K27 alterations (DMG, H3‐K27M), (2) diffuse hemispheric glioma with H3‐G34 mutations (DHG, H3G34M), and (3) diffuse pediatric‐type high‐grade glioma with wild‐type H3 and isocitrate dehydrogenase (pHGG, H3‐WT/IDH WT). Clinically, pHGGs are known for their poor outcomes and marked tumor heterogeneity. Despite this, the characteristics of the tumor microenvironment (TME) and the processes governing tumor cell lineage transitions remain incompletely understood. In this study, we used single‐cell RNA sequencing (scRNA‐seq) to analyze pHGG tumor cells (excluding infant‐type hemispheric gliomas). Through comprehensive bioinformatic approaches—including cell proportion analysis, Gene Ontology (GO) enrichment, metabolic activity inference via scMetabolism, proliferation gene scoring, stemness assessment by CytoTRACE2, SCENT, and pseudotime trajectory analysis with Monocle2—we thoroughly investigated the TME features and heterogeneity of these aggressive brain tumors. Our findings highlight the presence of oligodendrocyte precursor cell (OPC)‐like subpopulations, with epidermal growth factor receptor (*EGFR*)‐expressing OPC‐like cells emerging as a potential tumorigenic origin in diffuse midline gliomas due to their distinct stemness properties. Notably, platelet‐derived growth factor receptor alpha (*PDGFRA*)‐positive cells exhibit high specificity in DMG, suggesting greater diagnostic and therapeutic potential than *EGFR*. Next‐generation sequencing (NGS) and multiplex immunofluorescence analyses confirmed their distinct expression pattern, supporting PDGFRA as a key molecular marker. Moreover, OPC‐like cells at different differentiation states may drive lineage transitions in DMG. Together, these findings enhance our understanding of pHGG—especially DMG—and point to new avenues for targeted therapy.

## Graphical abstract

The graphical abstract will illustrate the key findings of our study, highlighting the OPC‐like subpopulations and their role in driving tumorigenic lineage transitions in pediatric high‐grade gliomas.

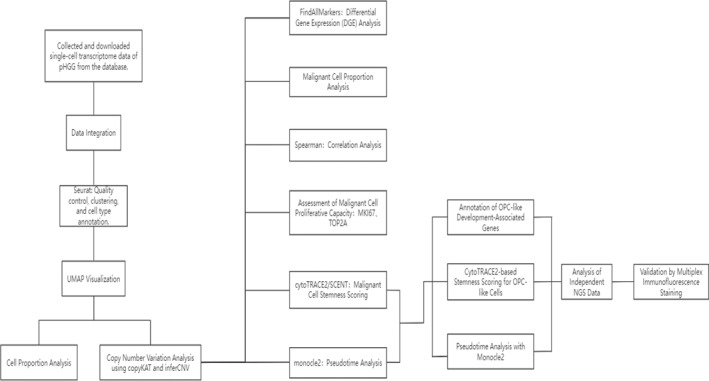

## 1 Introduction

The 2021 fifth edition of the WHO classification of tumors of the central nervous system introduced the category of “pediatric‐type diffuse high‐grade gliomas (pHGGs)”, emphasizing their distinct biological and clinical features compared to adult‐type diffuse high‐grade gliomas aHGG). This classification underscores the importance of studying pediatric gliomas as distinct entities [1]. pHGGs are associated with dismal clinical outcomes, with a median overall survival (OS) of 9–15 months [1]. The classification includes the following subtypes: (1) diffuse midline glioma, H3‐K27‐altered (DMG, H3‐K27M), (2) diffuse hemispheric glioma, H3 G34‐mutant (DHG, H3‐G34M), (3) diffuse pediatric‐type high‐grade glioma, H3‐wildtype and IDH‐wildtype (pHGG, H3‐WT/IDH WT), and (4) infant‐type hemispheric glioma [2, 3]. These tumors exhibit significant heterogeneity in their tumor microenvironment (TME) and cellular composition, which may contribute to their aggressive behavior and resistance to therapy [4].

Tumor heterogeneity is commonly categorized into classical heritable heterogeneity, tumor evolution with compositional diversity, and transient heterogeneity driven by the TME [5]. The evolution and heterogeneity of cancer have long been debated, particularly concerning tumor origins. It is hypothesized that tumors arise from cancer stem cell populations due to their intrinsic longevity, which increases their susceptibility to accumulating oncogenic mutations [6]. Furthermore, the TME plays a critical role in regulating the self‐renewal and differentiation of these cancer stem cells.

Advances in single‐cell RNA sequencing (scRNA‐seq) have significantly enhanced the ability to resolve tumor heterogeneity at single‐cell resolution. Recent studies in pediatric tumors, such as medulloblastoma [7], neuroblastoma [8], and ependymoma [9], have demonstrated the utility of scRNA‐seq for characterizing TME heterogeneity and tracing lineage transitions, ultimately enabling the inference of tumor origins.

Pediatric high‐grade gliomas (pHGGs) are currently recognized as neurodevelopmental disorders [10, 11]. However, the precise cellular origins remain poorly understood, and pHGGs exhibit profound heterogeneity. For instance, high‐grade gliomas (HGG) harboring H3.3 K27M mutations are thought to originate from glial progenitor cells, where the mutation impairing differentiation is along the glial lineage [12].

In this study, we leveraged publicly available scRNA‐seq datasets of pHGG to investigate the heterogeneity and characteristics of the TME. Additionally, we explored tumor cell lineage transitions to identify potential stem cells implicated in tumor origins. These findings were further validated using immunofluorescence analysis, offering insights into the biological mechanisms underlying pHGG progression and its cellular dynamics.

## 2 Method

### 2.1 Human Subjects and Ethical Considerations

The samples used in this study were anonymized and obtained with informed consent from the patients or their legal guardians, without any financial compensation (Approval No. 409, 2024, Clinical Research Ethics Committee).

### 2.2 Tumor Tissue Immunofluorescence Staining and Imaging

Tumor tissues were fixed in 4% paraformaldehyde, sectioned into 20 μm slices, and paraffin‐embedded. Antigen retrieval was performed using sodium citrate buffer (pH 6.0) at 95°C for 15 min, followed by blocking with 5% bovine serum albumin (BSA) in phosphate‐buffered saline (PBS) for 1 h at room temperature. The sections were incubated overnight at 4°C with primary antibodies: PDGFRα (1:1000; Cell Signaling, D1E1E; Danvers, MA, USA) and OLIG2 (1:1000; Cell Signaling, E6G6Q; Danvers, MA, USA), diluted in 1% BSA/PBS. Secondary antibodies (Alexa Fluor Plus 555 Goat anti‐Mouse IgG, A32727; Alexa Fluor Plus 488 Goat anti‐Rabbit IgG, A32731; Thermo Fisher Scientific, Waltham, MA, USA) were applied at a 1:1000 dilution for 1 h in the dark at room temperature. Nuclei were counterstained with DAPI.

Fluorescence signals were visualized using a Zeiss LSM780 confocal microscope (Carl Zeiss Microscopy GmbH, Oberkochen, Germany) with excitation at 488 nm (Alexa Fluor 488; Thermo Fisher Scientific, Waltham, MA, USA) and 555 nm (Alexa Fluor 555; Thermo Fisher Scientific, Waltham, MA, USA). Negative controls and omitting primary antibodies were included to ensure specificity.

### 2.3 Statistics and Reproducibility

We did not use statistical methods to set the sample size, and all data were included in the analysis. The experiments were nonrandomized, and neither data collection nor analysis was blinded to experimental conditions.

Statistical analysis was performed in R v.4.3.0. Numerical variables were compared using the Bonferroni test when appropriate. Single‐cell sequencing for each tumor was performed as a single replicate and a common practice in human studies due to the limited availability of tissue samples. To ensure reproducibility, at least three samples were collected from each anatomical site group of pHGG.

### 2.4 Cells Clustering and Doublet Removal

Cells were clustered using Seurat v4.3.0 [13]. Data were normalized, and 3000 highly variable genes were selected. Principal component analysis (PCA) was applied for dimensionality reduction, and clustering was performed using FindClusters, with results visualized via UMAP. Cells were filtered to have 200 to 12,000 genes, mitochondrial gene expression below 25%, and total RNA count below 50,000. Batch effects were corrected using Harmony (v1.2.0) on the first 100 principal components (PCs) [14].

We used DoubletFinder (v2.0.3) to identify and remove doublets from scRNA‐seq data [15]. The analysis involved defining 1 to 30 principal components and optimizing parameters using the BC metric. The doublet rate was estimated, and doublet detection was performed to enhance data accuracy.

### 2.5 Subtype‐Proportional Stratified Sampling

To minimize sampling bias due to group size imbalance, we performed five rounds of stratified subsampling based on the smallest group size. Subsampling was proportionally conducted across cell subtypes, with different random seeds used for each repetition to ensure stability. All downstream analyses were performed based on the stratified subsampling results.

### 2.6 Cell Subtype Proportion Analysis

The cluster ratio plot function was utilized to generate stacked bar charts displaying the proportion of different cell subtypes across groups, based on metadata. This analysis highlights the distribution of cell subtypes within various groups, using specific color codes for each subtype. To improve readability, *X*‐axis labels were rotated by 45°.

### 2.7 Identification of Malignant and Non‐Malignant Cells

We used the CopyKAT tool (v4.0.5) [16] to differentiate between malignant and nonmalignant cells. RNA expression matrices for each cell subtype were processed by group, and the CopyKAT algorithm computed copy number variations (CNVs). Results were integrated into the Seurat object. The predictions were visualized using UMAP plots.

### 2.8 Copy Number Variation Analysis

We used the inferCNV (v1.14.2) [17] to analyze copy number variations in single‐cell RNA sequencing data. Input parameters (grouping information, species type, Seurat object, cell type annotation, and reference cell types) were parsed using optparse. The gene position file was selected, target cell subpopulations were extracted, and the analysis was run with parameters including reference groups, output format, and clustering method.

### 2.9 Analysis of Gene Expression (*TOP2A*, *MKI67*, *EGFR* and *PDGFRA*)

Using single‐cell RNA sequencing data, this study investigated the expression patterns of TOP2A and MKI67 in malignant cells. Box plots with jitter points were generated after filtering nonzero expression values to compare expression levels across DMG, DHG, and H3WT groups. Similarly, based on stratified sampling results, *EGFR* and *PDGFRA* expression features were further analyzed in OPC‐like cells of DMG. Statistical significance was assessed using the Wilcoxon rank‐sum test with Bonferroni correction, and significant *p*‐values were annotated on the plots.

### 2.10 Gene Enrichment Analysis of Malignant Cell Subtypes

Gene enrichment analysis was conducted using COSG (v0.9.0) [18] on malignant cell subtypes, electing the top 100 genes for subsequent analyses. For the OPC‐like subtype, the analysis was extended to the top 500 genes, providing valuable insights for functional studies.

### 2.11 KEGG and GO Pathway Enrichment Analysis

KEGG and GO pathway enrichment analyses [19] were performed using the selected genes to uncover critical biological processes and signaling pathways of malignant cell subtypes, offering a deeper understanding of their functional roles.

### 2.12 Metabolic Characterization of Malignant Cell Subtypes

scMetabolism (v0.2.1) [20] was utilized to analyze the metabolic characteristics of malignant cell subtypes, randomly sampling 85% of the cells. AUCell was applied to compute the average metabolic scores for KEGG pathways across DMG, DHG, and H3WT groups. The metabolic features were visualized using heatmaps, highlighting significant metabolic differences among malignant cell subtypes in distinct pHGG types.

### 2.13 Evaluation of Differentiation Potential Using CytoTRACE2

CytoTRACE2 (v1.0.0) [21] was used to analyze the differentiation potential of malignant cell subtypes. Stemness scores were calculated for each subtype and visualized using UMAP plots and box plots. Statistical significance was assessed with Wilcoxon tests, providing insights into the biological characteristics of malignant cell subtypes.

### 2.14 Stemness Evaluation Using SCENT

In this study, SCENT (v1.0.3) [22] algorithm was used to assess the stemness potential of pHGG and OPC‐like cells. Gene expression data were normalized by the relative counts (RC) method based on counts per million (CPM), followed by log_2_ (x+ 1.1) transformation. Genes with unique Entrez IDs were selected for analysis. CCAT was calculated based on the human protein interaction network and visualized by box plots.

### 2.15 Pseudotime Trajectory Analysis of Tumor Cells

Pseudotime trajectories of tumor cells were analyzed using Monocle2 (v4.0.5) [23]. Expression matrices and cell annotations were extracted from Seurat objects to construct a Monocle object. After preprocessing and filtering, marker genes were identified via differential gene testing. Pseudotime trajectories, state distributions, and cell subtypes were visualized to reveal the dynamic changes and biological significance of various tumor cell populations.

### 2.16 Sample Handling, DNA Extraction and Next‐Generation Sequencing

Twelve patient samples were analyzed. For each, five 5‐μm formalin‐fixed paraffin‐embedded (FFPE) sections and 10 mL of peripheral blood were collected. Plasma and leukocytes were separated by centrifugation, and genomic DNA was extracted using the AllPrep DNA/RNA Mini Kit (Qiagen,Hilden, Germany), with leukocyte DNA as the germline control. DNA quality and fragment size were assessed via Bioanalyzer (Agilent Technologies, Santa Clara, CA, USA). Libraries were prepared using the KAPA Hyper Prep Kit and captured with a customized 919‐gene panel (Onco Panscan, Genetron Health, Beijing, China) (Table S1).

### 2.17 Genomic Data Processing and Mutation Analysis

Sequencing data were processed with Trimmomatic (v0.36) [24], mapped to GRCh37/hg19 using BWA (v0.7.10) [25] and duplicates removed with PICARD (v2.2.1). Local realignment and BQSR were performed with GATK (v3.5) [26]. Somatic SNVs/InDels were called using Mutect (v3.1) [27] and Strelka (v2.9.2) [27], CNVs with ADTEx (http://adtex.sourceforge.net/), and SVs with CREST [28, 29].

QC: tumor ≥ 500 × depth, ≥ 90% coverage at ≥ 140 ×; leukocyte ≥ 250 × depth, ≥ 90% coverage at ≥ 10 ×. SNV/InDel criteria: ≥ 7 supporting reads, VAF ≥ 1% (hotspot), ≥ 5% (nonhotspot). CNV: amplification ≥ 3 CN, loss ≤ 1.55 CN. Fusion mutations: ≥ 1% frequency.

Mutations were annotated with VEP (v92) [30].and filtered using 1000 Genomes [31], ESP, ExAC [32] and gnomAD. IGV [33]was used for artifact filtering. The high‐frequency mutated gene landscapes in DMG cohort were visualized by R package ComplexHeatmap v2.18.0.

## 3 Results

### 3.1 Integrative Analysis of Genetic Mutations, Clinical Features, and Cellular Heterogeneity in pHGGs

**FIGURE 1:**
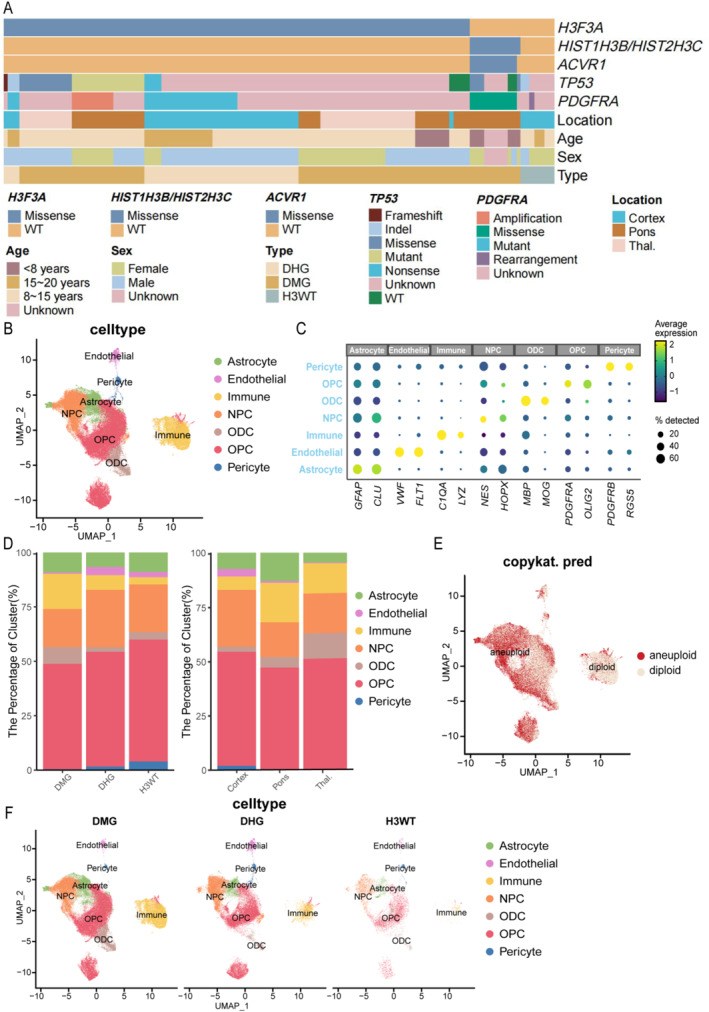
Single‐cell transcriptomic analysis of the pHGG cohort. (A) Clinical and molecular characteristics of the cohort, summarizing data from 32 pHGG samples. (B) UMAP visualization illustrating the distribution of distinct cell types. (C) Expression patterns of representative marker genes in pHGG. (D) Proportional distribution of cell types stratified by pHGG subtype and anatomical location. (E) CopyKAT analysis distinguishing malignant from nonmalignant cell populations, visualized using UMAP. (F) UMAP visualization of cell populations grouped by pHGG subtype. C1QA, complement C1q A chain; CLU, clusterin; FLT1, fms related receptor tyrosine kinase 1; GFAP, glial fibrillary acidic protein; HOPX, HOP homeobox; LYZ, lysozyme; MBP, myelin basic protein; MOG, myelin oligodendrocyte glycoprotein; NES, nestin; OLIG2, oligodendrocyte transcription factor 2; PDGFRA, platelet‐derived growth factor receptor alpha; PDGFRB, platelet‐derived growth factor receptor beta; RGS5, regulator of G protein signaling five; Thal, thalamus; UMAP, Uniform Manifold Approximation and Projection; VWF, von Willebrand factor.

We conducted single‐cell transcriptomic analysis on tumors from 32 pediatric and adolescent patients with pHGGs, including 18 DMG H3‐K27M, 10 DHG H3‐G34R/V, and 4 pHGG WT cases, using data from GSE210568. Tumors were selected based on histone mutation status and were sourced from three distinct anatomical locations: the pons (*n* = 13), thalamus (*n* = 4), and cerebral cortex (*n* = 15) (Figure 1A). The median age of the patients was 11.5 years (range: 3.4–20 years). To explore additional mutations, we incorporated whole‐exome and targeted exome sequencing data [12]. Mutations in *TP53* and *PDGFRA* were found consistently across all clinical and anatomical subgroups, regardless of pHGG subtype, age, or location. However, alterations in *HIST1H3B/HIST2H3C* were rarely detected in pediatric tumors, consistent with previous studies [1, 34, 35].

At a resolution of 0.3, we identified 153,369 cells, which were classified into multiple populations, including astrocytes, neural progenitor cells (NPCs), oligodendrocytes (ODCs), oligodendrocyte progenitor cells (OPCs), endothelial cells, and pericytes (Figure 1B,C). The proportions of these cell types varied significantly across different pHGG subtypes, underscoring the high degree of tumor heterogeneity (Figure 1D,F). Immune cells were more prevalent in DMG H3K27M tumors, whereas neural progenitor cells predominated in DHG H3G34R/V. Notably, pericytes were exclusively detected in DHG H3G34R/V and pHGG WT tumors. Moreover, the cellular composition of pHGG varied across anatomical locations. CopyKAT analysis (Figure 1E) indicates that numerous malignant cells are present in astrocytes, NPCs, ODCs, and OPCs. This finding is consistent with the results from inferCNV analysis, suggesting that these cell populations may serve as the primary origin of tumor cells in pHGGs.

Overall, our cohort captures the key clinical and molecular features of pHGG, emphasizing the considerable cellular heterogeneity among tumor subtypes and underscoring the complex biological nature of this disease. Subsequent analyses will further investigate nongenetic factors and explore the heterogeneity across different pHGG subtypes.

### 3.2 Malignant Cell Heterogeneity in pHGG Subtypes

**FIGURE 2:**
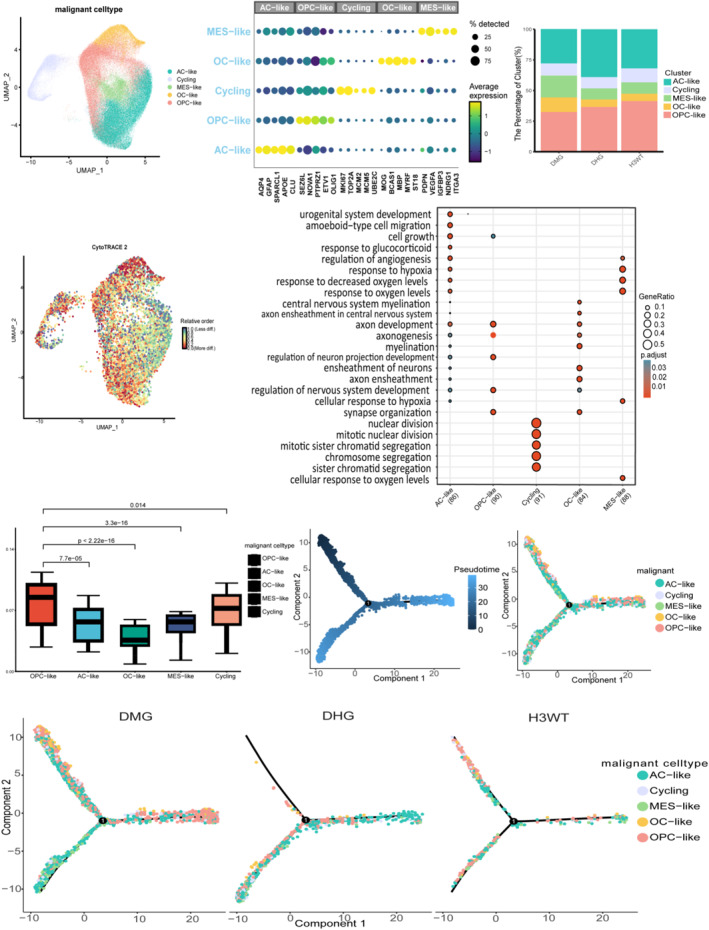
Analysis of malignant tumor cell subpopulations. (A) UMAP visualization showing the reclustering of malignant tumor cell subpopulations into five distinct groups. (B) Expression patterns of representative marker genes in pHGG. (C) Cell proportion diagram illustrating the distribution of malignant tumor cell subpopulations across pHGGs. (D) SCENT analysis of tumor cell stemness potential, presented as box plots. (E) Stemness scores mapped onto a UMAP plot. (F) Gene Ontology (GO) enrichment analysis of malignant tumor cell subpopulations. (G, H) Pseudotime analysis of malignant tumor cell subpopulations. (I) Differentiation trajectories of malignant tumor cells across different pHGG subtypes. AC‐like: Astrocyte‐like cells; Cycling: Cycling cells; MES‐like: Mesenchymal‐like; OC‐like: Oligodendrocyte‐like cells; OPC‐like: Oligodendrocyte Precursor Cell‐like cells. APOE, Apolipoprotein E; AQP4, Aquaporin 4; BCAS1, Breast Carcinoma Amplified Sequence 1; CLU, Clusterin; ETV1, ETS Variant 1; GFAP, Glial Fibrillary Acidic Protein; IGFBP3, Insulin‐Like Growth Factor Binding Protein 3; ITGA3, Integrin Subunit Alpha 3; MBP, Myelin Basic Protein; MCM2, Minichromosome Maintenance Complex Component 2; MCM5, Minichromosome Maintenance Complex Component 5; MKI67, Marker of Proliferation Ki‐67; MOG, Myelin ligodendrocyte Glycoprotein; MYRF, Myelin Regulatory Factor; NDRG1, N‐Myc Downstream Regulated Gene 1; NOVA1, RNA‐binding Protein Nova‐1; OLIG1, Oligodendrocyte Transcription Factor 1; PDPN, Podoplanin; PTPRZ1, Protein Tyrosine Phosphatase Receptor Type Z1; SEZ6L, Seizure‐related 6 Homolog Like; SPARCL1, SPARC‐like Protein 1; ST18, Suppressor of Tumorigenicity 18; TOP2A, DNA Topoisomerase II Alpha 170kDa; UBE2C, Ubiquitin Conjugating Enzyme E2 C; VEGFA, Vascular Endothelial Growth Factor A.

In this study, we aimed to characterize and compare the transcriptional heterogeneity within our cohort of pHGG subtypes (Figure 1A). Based on stratified analysis, we compared the biological characteristics of DMG across different anatomical locations and found that DMG from various anatomical sites exhibited highly similar biological features. Through differential gene expression analysis using FindAllMarkers and integrated approaches [12], we identified tumor cells displaying characteristics of active cycling, oligodendrocyte precursor cell‐like (OPC‐like), astrocyte‐like (AC‐like), oligodendrocyte‐like (OC‐like), and mesenchymal‐like (MES‐like) phenotypes (Figure 2A–C). We selected five key cell markers for presentation (Figure 2B). Notably, MES‐like and OC‐like cell populations were more abundant in DMG than in DHG and H3WT gliomas, whereas AC‐like cells were more prevalent in DHG, and OPC‐like cells were most abundant in H3WT gliomas. The proportion of cycling cells remained consistent across all three pHGG subtypes (Figure 2C). Intriguingly, MES‐like features previously identified in glioblastoma (GBM) [36, 37] were also observed in our cohort, particularly within underexplored clinical and anatomical subgroups in this expanded dataset. Malignant OPC‐like cells were present across all three pHGG subtypes (Figure 2D) but varied in proportion, reflecting the high degree of intertumoral heterogeneity.

To further explore the tumor heterogeneity, we conducted Gene Ontology (GO) enrichment analysis on the top 500 differentially expressed genes (Figure 2F). GO analysis revealed that both AC‐like and MES‐like cells were enriched in hypoxia‐related pathways, with AC‐like cells uniquely enriched for pathways related to cell growth and migration, glucocorticoid response, and axon development. In contrast, OC‐like cells were enriched in neural development pathways, such as axonogenesis and myelination. Cycling cells were predominantly enriched in mitosis‐related pathways. Proliferation‐related genes such as *MKI67* and *TOP2A* (Supporting Information S1: Figure S3F–G) revealed that the proliferative capacity of the same malignant cell type varied among different pHGG subtypes. Malignant cells in H3WT exhibited slightly higher proliferative capacity compared to those in DMG and DHG. Furthermore, metabolic analysis (Supporting Information S1: Figure S3A–E) revealed significant metabolic differences among malignant cell types in different pHGG subtypes, further emphasizing the extensive heterogeneity of pHGG.

We next assessed stemness across these cells using CytoTRACE2 and SCENT (Figure 2C,D). OPC‐like cells consistently exhibited the highest stemness and differentiation potential across all three pHGG subtypes (Figure 2E,F). To explore potential lineage transitions in pHGG, we performed pseudotime trajectory analysis using Monocle2 to model cellular differentiation dynamics [38, 39](Figure 2G–I). In the early stages of tumor differentiation, a mixture of tumor cell types, predominantly OPC‐like and AC‐like cells, was observed. When analyzed by individual pHGG subtype, distinct differentiation origins emerged. In DMG, OPC‐like cells were identified as the primary initiators of differentiation, following a more continuous trajectory, in line with previous studies [40, 41]. In contrast, AC‐like cells were more prominent during the early differentiation stage in DHG.

In conclusion, our findings highlight significant heterogeneity among pHGG subtypes, with OPC‐like cells potentially serving as the origin of tumor differentiation in DMG.

### 3.3 Distinct Roles of EGFR and PDGFRA in OPC‐Like Cell Differentiation and Tumor Heterogeneity in pHGGs

In this study, we focused on the analysis of OPC‐like cell subpopulations identified from scRNA‐seq data. To minimize anatomical biases, we first compared DMG samples from different brain regions, such as the pons and thalamus. Their transcriptional profiles were largely concordant, suggesting that anatomical location had limited impact on our subsequent analyses.

OPC‐like cells were identified by their high expression of canonical OPC marker genes, such as *OLIG1* and *PTPRZ1* (Figure 2B). Using the Seurat FindAllMarkers function, we identified four OPC‐like subpopulations (OPC‐1 to OPC‐4; Figure 3A,B). Notably, although OPC‐1 and OPC‐2 are both enriched for specific pathways, they displayed distinct developmental gene expression profiles and positions along pseudotime, suggesting divergent functional states. OPC‐2 and OPC‐3 were particularly enriched in DMG samples and showed no dependency on anatomical locations (Figure 3I). OPC‐3 shows high ribosomal‐gene expression, a hallmark of early OPCs in mouse and human development [12, 42].

FIGURE 3The role of OPC‐like cell subpopulations in pHGG cellular lineage transitions. (A) UMAP visualization showing the reclustering of OPC‐like cells into four distinct subpopulations. (B) Expression patterns of representative marker genes in OPC‐like cells, with the top five genes highlighted. (C) Stemness scores of OPC‐like cell subpopulations, presented as box plots. (D) Stemness scores mapped onto a UMAP plot. (E) Gene Ontology (GO) enrichment analysis of OPC‐like cell subpopulations. (F, G) Pseudotime trajectory analysis of OPC‐like cell subpopulations. (H) Pseudotime trajectory analysis of OPC‐like cell subpopulations across different pHGG subtypes. (I) Proportional distribution of OPC‐like cells across pHGG subtypes and anatomical locations in DMGs. (J) Expression patterns of key marker genes associated with oligodendrocyte precursor cell (OPC) development. The result of 919‐gene panel sequencing in 12 DMG samples. The plot shows high‐frequency mutations in each sample, with mutation types indicated by different colors. (L,M) The expression of EGFR and PDGFRA in OPC‐like cells of DMG, visualized by FeaturePlot. (N) Box plots show EGFR and PDGFRA expression in OPC‐like across various DMG regions. (O) OPC‐like cells positive for PDGFRA in DMG patient samples. OLIG2 is stained in red, PDGFRA in green, and DAPI in blue.

**Figure:**
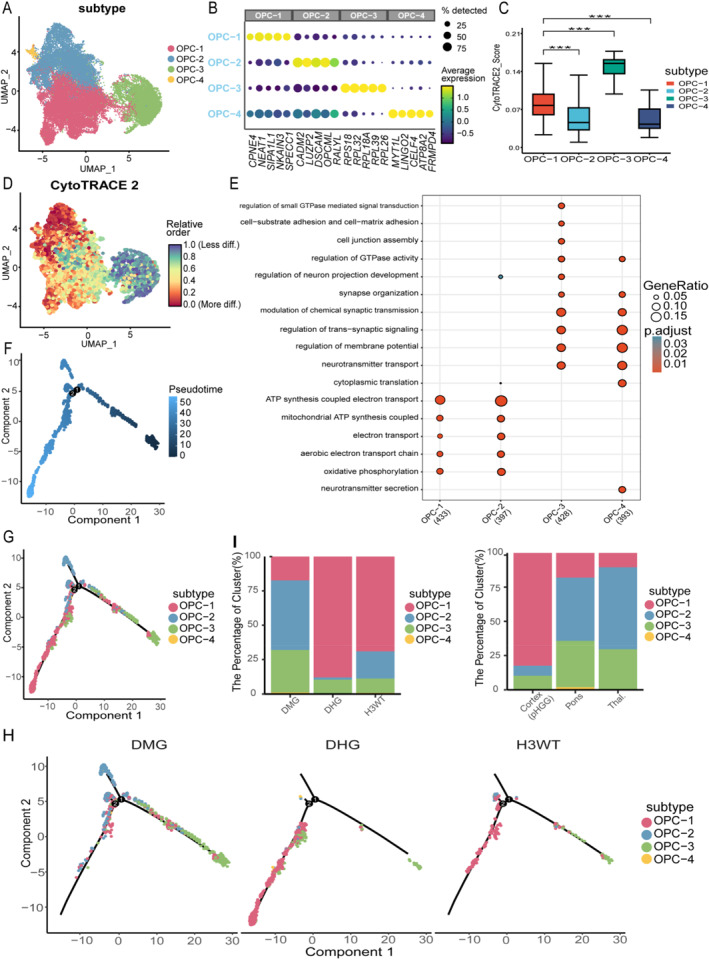

**Figure:**
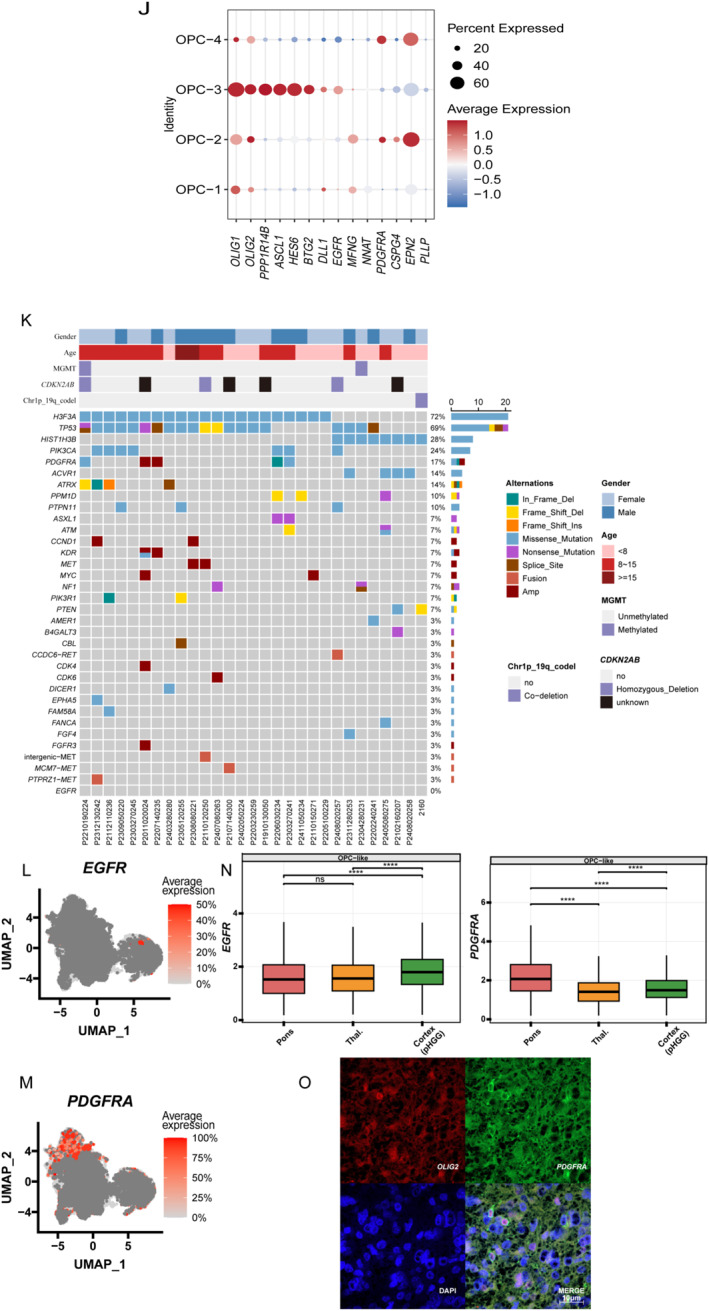

Gene Ontology (GO) enrichment analysis further revealed functional heterogeneity among the four subgroups (Figure 3E). OPC‐1 and OPC‐2 were enriched in energy metabolism pathways, including mitochondrial ATP synthesis and oxidative phosphorylation. In contrast, OPC‐3 and OPC‐4 were enriched in neurodevelopmental pathways, such as neuron projection development, synaptic signaling, and membrane potential regulation. OPC‐3 was uniquely enriched in cell adhesion and small GTPase signaling pathways that were absent in OPC‐4, suggesting potential differences in migratory capacity. These findings support pronounced functional heterogeneity among OPC‐like cells in DMG.

To investigate the distribution of OPC‐like subpopulations across pHGG subtypes and anatomical regions, we generated proportion plots (Figure 3I). OPC‐2 and OPC‐3 were significantly enriched in DMG and displayed consistent patterns across brainstem and thalamic samples, highlighting their potential relevance to DMG biology. This enrichment was not observed in DHG or H3WT, suggesting that OPC‐2 and OPC‐3 may be DMG‐specific populations.

CytoTRACE2 and SCENT both assign the highest stemness score to OPC‐3 (Figure 3C,D) and validated the results using SCENT analysis. Both analyses identified OPC‐3 as having the highest stemness potential, whereas OPC‐2 and OPC‐4 scored the lowest, suggesting that OPC‐3 may represent an earlier developmental state [43].

Pseudotime analysis positions OPC‐3 at the root of the trajectory, implying a tumor‐initiating role in DMG (Figure 3F–H) [44]. The trajectory was more continuous and focused in DMG, with a noticeable “differentiation bottleneck” at the OPC‐2 stage, which was not seen in other pHGG subtypes (Figure 3H). This bottleneck aligns with the low stemness scores of OPC‐2 and may reflect its restricted differentiation potential, possibly due to driver gene influences. Together, these findings suggest that OPC‐3 may be the origin of malignant transformation in DMG.

We further examined developmental markers of OPCs from previous studies [45, 46] and found that *EGFR* and *PDGFRA* marked distinct stages along the OPC‐like trajectory (Figure 3J). Specifically, EGFR, a key marker of pre‐OPC, was predominantly expressed in OPC‐3, consistent with a “pre‐OPC” identity, whereas PDGFRA was highly expressed in OPC‐2 and OPC‐4, marking a more mature OPC state. This expression pattern is consistent with previous reports showing that EGFR is activated early in OPC development [47, 48, 49], whereas PDGFRA characterizes more differentiated OPCs [46, 47]. These observations led us to investigate their potential as molecular drivers.

To assess clinical relevance, we analyzed mutations and expression levels of *EGFR* and *PDGFRA* (Table S1). In 12 DMG samples subjected to next‐generation sequencing (NGS), *PDGFRA* mutations occurred in 17%, whereas *EGFR* mutations were absent (Figure 3L), suggesting higher mutational relevance of *PDGFRA*. FeaturePlot analysis revealed that PDGFRA was more highly expressed than EGFR in DMG OPC‐like cells, particularly in OPC‐2 and OPC‐4 (Figure 3M). Given that these two subpopulations are abundant in DMG and express high *PDGFRA*, we performed multiplex immunofluorescence on five DMG tumors. Each sample was stained in technical triplicates, and representative images with optimal signal quality were presented (Figure 3O). Immunostaining confirmed the presence of *PDGFRA*‐positive OPC‐like cells at the tissue level.

Overall, PDGFRA exhibited greater specificity than EGFR in terms of expression, mutation frequency, and subpopulation abundance, supporting its potential as a therapeutic target. This conclusion is reinforced by further scRNA‐seq analyses. *PDGFRA* mutations were restricted to OPC‐like cells and rare in AC‐like or MES‐like populations, suggesting a subtype‐specific role in driving malignancy [50]. Notably, PDGFRA expression was significantly higher in pontine DMGs compared to thalamic or spinal samples, indicating spatial specificity and potential correlation with tumor aggressiveness [50].

Importantly, our findings are consistent with prior studies. G. J. Rahme et al. reported in cells that epigenetic activation of PDGFRA in OPCs—via disruption of CCCTC‐binding factor (CTCF) insulation—could cooperate in gliomagenesis, underscoring its role in tumor initiation [50]. Additionally, recent clinical data suggest that PDGFRA‐targeted therapy shows promising responses in subsets of pHGG patients [51].

In conclusion, our integrative genomic and transcriptomic analysis highlights *PDGFRA* as a potential therapeutic target, owing to its high mutation frequency, elevated expression in specific OPC‐like subpopulations, and tumor‐driving potential.

## 4 Discussion

Emerging evidence suggests that pHGGs originate from aberrant neurodevelopmental processes rather than representing mere recapitulations of adult GBMs [10, 11]. Integrating scRNA‐seq, next‐generation sequencing (NGS), and multiplex immunofluorescence, our study provides new insights into the potential roles of OPC‐like subpopulations—particularly those expressing EGFR and PDGFRA—in tumor initiation, progression, and lineage transitions. Although prior studies have implicated glial precursor‐ or stem‐like populations as the likely origins of pediatric gliomas [44, 45, 46, 47, 52], our integrative approach refines the understanding of OPC‐like subgroups in DMG and other pHGG subtypes. We demonstrate that PDGFRA outperforms EGFR in terms of expression, mutation frequency, and subgroup specificity, indicating its greater therapeutic potential, especially in DMG.

### 4.1 Comparison With Existing Studies on Tumor Heterogeneity

Tumor heterogeneity can arise from genetic alterations, clonal evolution, and transient states influenced by the microenvironment [5]. Our scRNA‐seq data reflect these dimensions within pHGG, revealing OPC‐like cells with stable lineage traits alongside dynamic populations shaped by local cues such as hypoxia or cytokine gradients, mirroring plasticity seen in adult GBM [37]. In pediatric tumors, this plasticity may be more pronounced due to the developmental context, posing challenges for targeted therapies.

### 4.2 OPC‐Like Cells in pHGG Tumorigenesis

We identified EGFR‐ and PDGFRA‐expressing OPC‐like populations with varying degrees of stemness and lineage commitment. Mechanistically, these cells may lie at the core of pHGG tumorigenesis, particularly in DMG. Notably, EGFR^+^ OPC‐like cells maintained stem‐like properties and showed a more continuous differentiation trajectory in DMG, suggesting a role as potential drivers of tumorigenesis. These findings build on earlier work implicating glial precursors in H3K27M gliomas [23, 24, 25, 26, 30, 44, 45, 46, 47, 52] and highlight the EGFR pathway's possible role in sustaining self‐renewal and blocking differentiation.

The existence of multiple OPC‐like states in pHGG aligns with current views of glioma cells occupying diverse lineage hierarchies [36, 37]. In adult GBM, scRNA‐seq reveals tumor cells distributed along trajectories ranging from AC‐like to OPC‐like states [36]. Despite distinct oncogenic drivers (e.g., histone mutations in children vs. IDH mutations or EGFR amplifications in adults), both harbor OPC‐like compartments, underscoring a shared tumor‐promoting potential within the glial lineage [5, 6]. Importantly, our data indicate that pediatric tumors—especially DMG—contain unique OPC subgroups shaped by anatomical and epigenetic contexts [11].

### 4.3 PDGFRA‐Expressing OPC‐Like Cells and Therapeutic Implications

PDGFRA‐high OPC‐like cells are enriched in DMG, consistent with reports of frequent PDGFRA alterations (amplifications or mutations) in pediatric brainstem gliomas [53] Given PDGFRA's role in the PI3K/AKT/mTOR pathway, it represents a promising therapeutic target, particularly as several PDGFRA inhibitors which are already in clinical development. Although current trials in pHGG show limited efficacy, our findings suggest that PDGFRA‐high OPC‐like cells could be the most responsive subpopulation [53].

However, the clinical validation of PDGFRA as a biomarker in pHGG/DMG remains limited, especially in terms of survival analysis or ROC curve performance. This reflects the relative infancy of research under the WHO 2021 classification and the scarcity of prognostic or treatment response data in public datasets [33]. Still, in PDGFRA‐high DMGs, targeting this receptor may hold promise, as *PDGFRA* amplification is associated with poorer prognosis [53, 54, 55].

Recent studies have highlighted the oncogenic role of PDGFRA signaling in glioma. A 2025 *Cancer Cell* study reported radiographic responses in *PDGFR*A‐mutant pHGG patients treated with the inhibitor avapritinib [51]. A 2023 *Cell* study revealed that *PDGFRA*‐driven gliomagenesis can arise via enhancer hijacking due to CTCF insulator loss at the *PDGFRA* locus [50]. PDGFRA is also a validated target in other solid tumors, such as gastrointestinal stromal tumor (GIST), where *PDGFRA‐D842V* mutations confer high sensitivity to avapritinib (objective response rate (ORR) up to 91%) [56].

In our study, we systematically characterized OPC‐like heterogeneity in pHGG, revealing significantly higher *PDGFRA* expression in DMG, especially in pontine tumors. UMAP clustering identified four OPC‐like subgroups (from OPC‐1 to OPC‐4), with OPC‐2 and OPC‐3 enriched in DMG and displaying high stemness, migration potential, and enrichment in neurodevelopmental and small GTPase pathways, suggesting key roles in progression (Figure 3) [12, 42, 44]. CytoTRACE and pseudotime analyses indicate that OPC‐3 may represent a tumor‐initiating population, whereas OPC‐2 may reflect a “differentiation‐stalled” state specific to DMG (Figure 3H) [43, 44].

*PDGFRA* expression is mainly localized in OPC‐2 and OPC‐4, with a mutation rate of 17% in DMG, compared to low *EGF*R expression and mutation frequency, further supporting *PDGFRA* as a subgroup‐specific target [51]. This aligns with the mechanism proposed by G. J. Rahme et al., where enhancer activation at the *PDGFRA* locus drives tumorigenesis via OPC‐specific programs [50].

Future efforts will include multicenter clinical cohorts to evaluate the diagnostic and prognostic value of *PDGFRA* in pHGG using Cox and ROC analyses [50], as well as functional studies using *PDGFRA*‐mutant organoids and orthotopic models to explore its role in proliferation and therapy resistance. Given avapritinib's activity, improving its CNS penetration and combining it with radiotherapy or immunotherapy are promising directions [56].

In conclusion, this study provides compelling evidence that OPC‐like subgroups are key contributors to the heterogeneity and progression of pHGG, especially in DMG. Using scRNA‐seq, next‐generation sequencing (NGS), and multiplex immunostaining, we identified at least two functionally relevant OPC‐like populations characterized by *EGFR* or *PDGFRA* expressions, each following distinct differentiation trajectories. These findings align with developmental models of pediatric brain tumors [10, 11]. Compared to *EGFR*, *PDGFRA* shows greater targeting potential based on its higher expression, mutation rate, and enrichment in specific subgroups, supporting its prioritization as a therapeutic target in DMG.

## Author Contributions

Tian Tian performed all data analyses. Chong Huang performedthe immunofluorescence staining. Lusheng Li conceptualized the study. Lusheng Li and Tian Tian wrote the paper.

## Ethics Statement

The present study has been approved by the Ethics Committee of Chongqing Medical University Affiliated Children's Hospital (Approval No. 409, 2024, Clinical Research Ethics Committee). The immunofluorescence‐stained samples and single‐cell transcriptomic data from the GEO database used in this research adhere to ethical standards.

## Conflicts of Interest

The authors declare no conflicts of interest.

## Data Citation

Jessa S., Mohammadnia A., Harutyunyan A.S., Hulswit M., Varadharajan S., Lakkis H., Kabir N., Bashardanesh Z., Hébert S., Faury D., Vladoiu M.C., Worme S., Coutelier M., Krug B., Faria Andrade A., Pathania M., Bajic A., Weil A.G., Ellezam B., Atkinson J., Dudley R.W.R., Farmer J.P., Perreault S., Garcia B.A., Larouche V., Blanchette M., Garzia L., Bhaduri A., Ligon K.L., Bandopadhayay P., Taylor M.D., Mack S.C., Jabado N., Kleinman C.L.; 2022; K27M in canonical and noncanonical H3 variants occurs in distinct oligodendroglial cell lineages in brain midline gliomas; Gene Expression Omnibus (GEO); DOI 10.1038/s41588‐022‐01205‐w.

## Supporting information


Supporting Information S1

**Table S1**: Summary of 12 DMG samples subjected to targeted next‐generation sequencing using a customized 919‐gene capture panel.

## Data Availability

The data that support the findings of this study are openly available in the Gene Expression Omnibus (GEO) at https://www.ncbi.nlm.nih.gov/geo/query/acc.cgi%20acc=GSE210568, reference number GSE210568 and available upon request from the corresponding author. The tissue samples are not publicly available due to privacy or ethical restrictions.
