# Supplementary material for: Single‐Cell Profiling of Pediatric High‐Grade Gliomas Reveals OPC‐Like Subpopulations Driving Tumorigenic Lineage Transitions
Source: Pediatr Discov. 2025 Sep 24;3(3):e70027. doi: 10.1002/pdi3.70027 (PMC12483302; doi:10.1002/pdi3.70027)
Supplement: Supplementary file 1 — Supporting Information S1 [file PDI3-3-e70027-s001.docx]

# Supplymentary Ⅰ

## Supplementary Figure 1


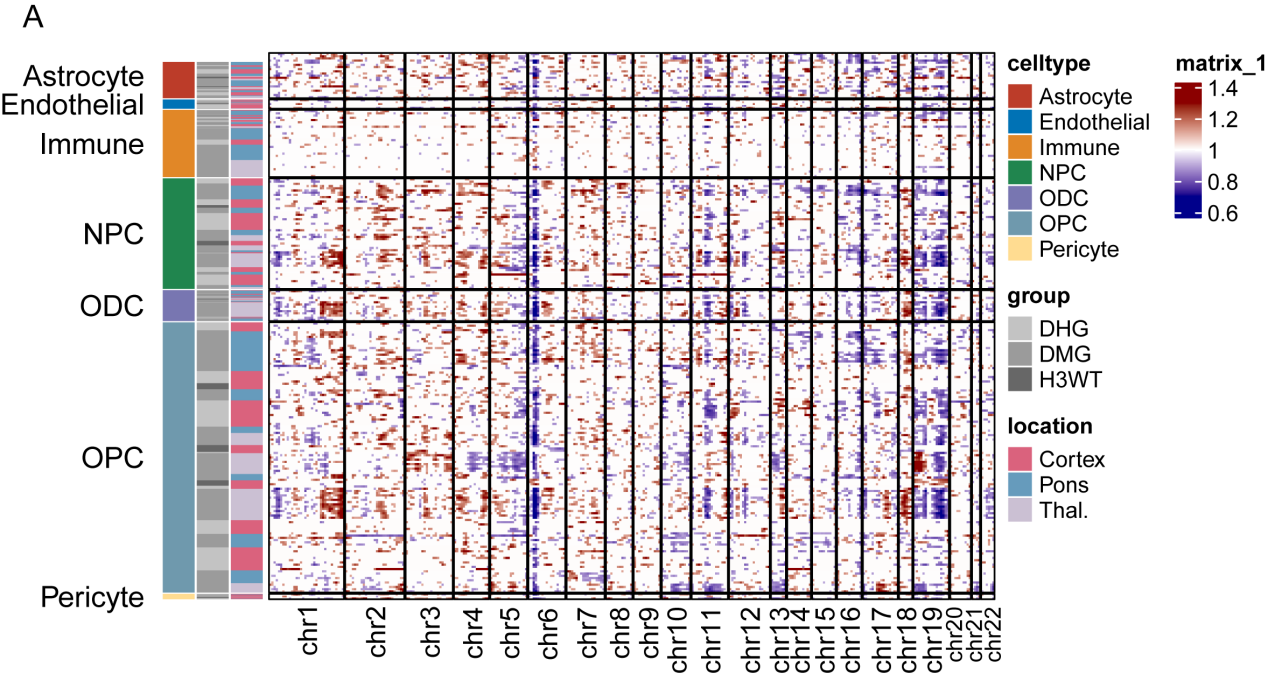


****Supplementary Figure 1:inferCNV Analysis of Copy Number Variations in pHGG Cells****

1. **inferCNV analysis shows that in pHGG, Astrocyte, NPC, OPC, and ODC exhibit significantly higher copy number variations than immune cells.**

## Supplementary Figure 2


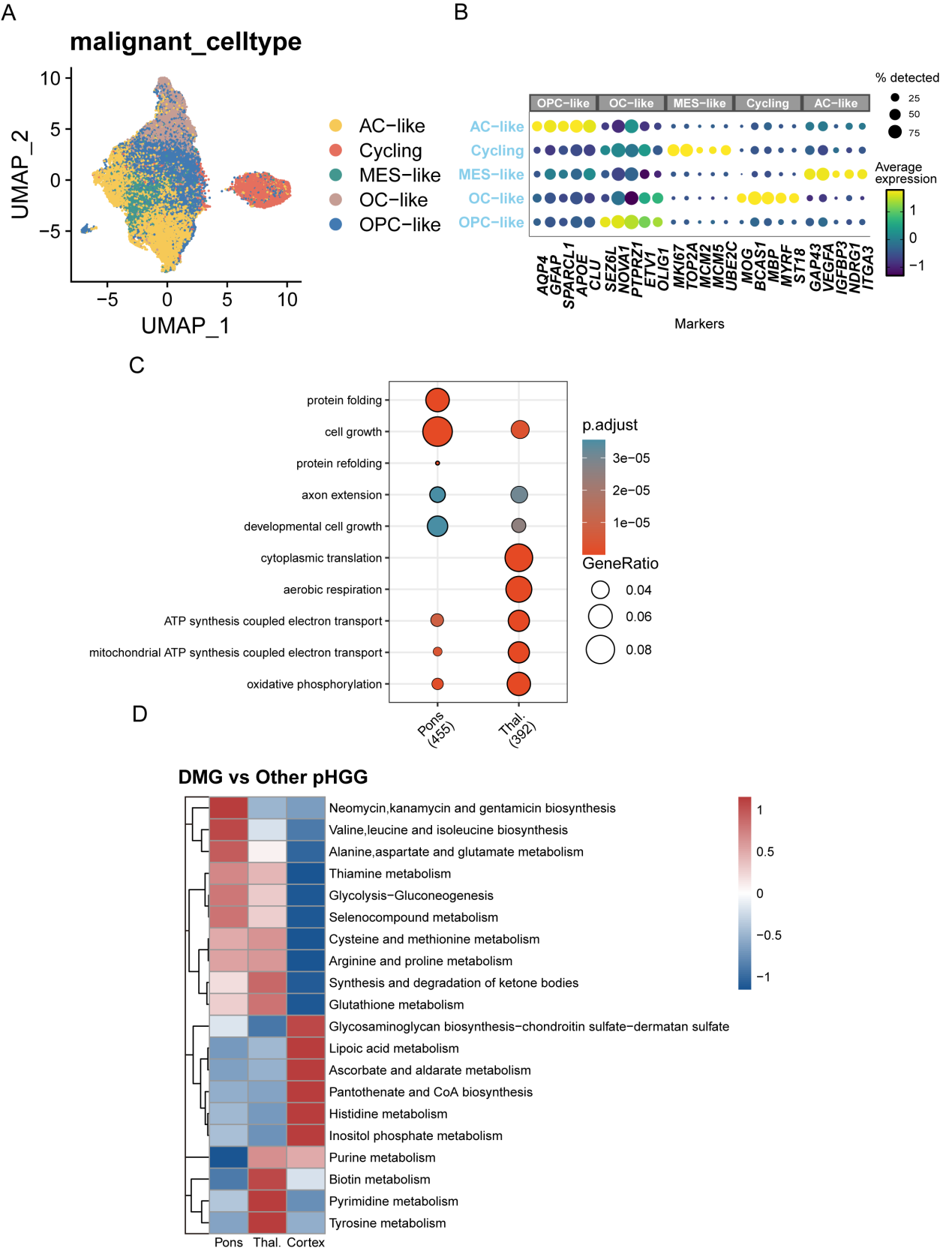


**
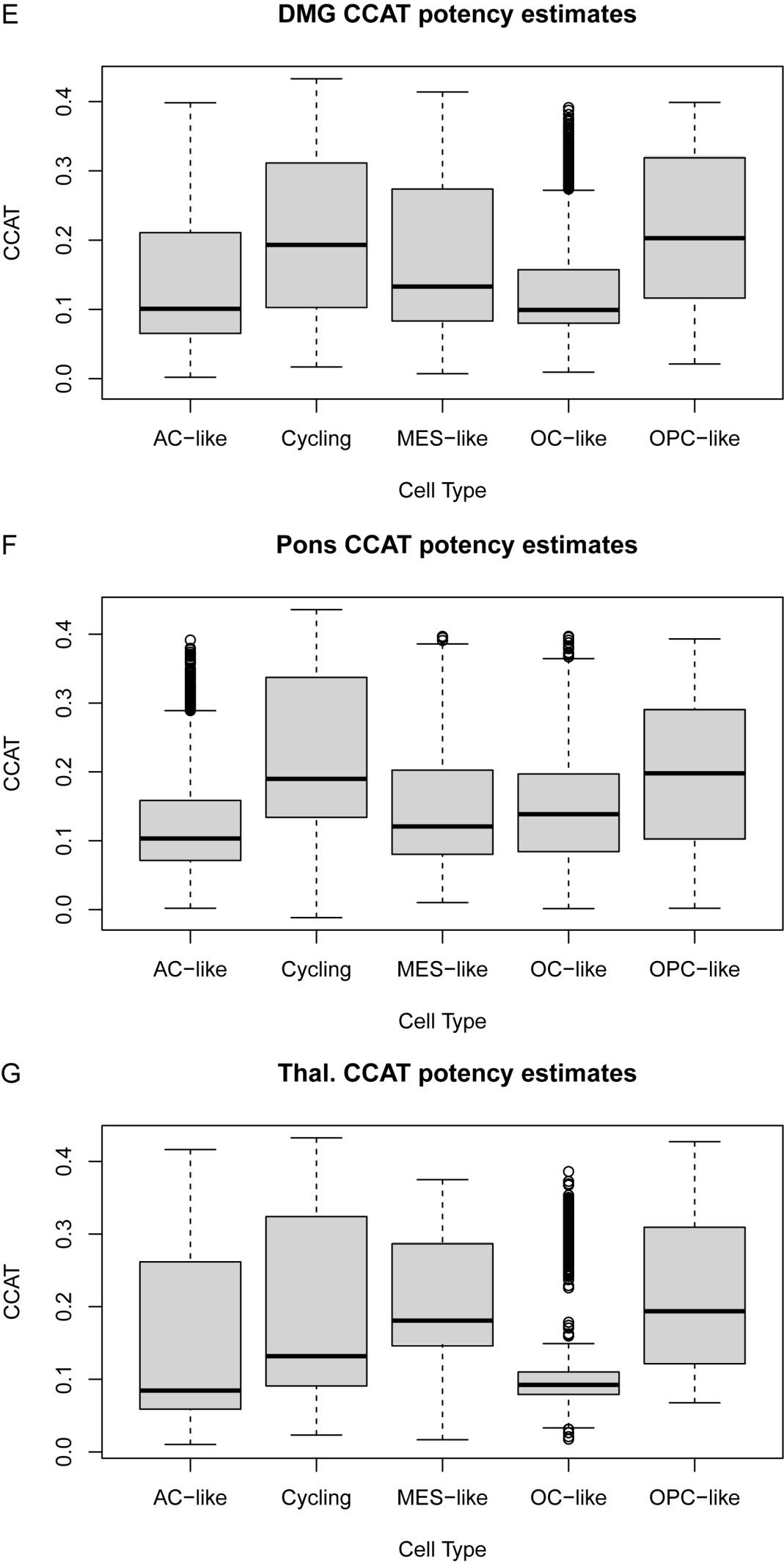
**

****Supplementary Figure 2:Comparative analysis of DMGs located in the pons and thalamus****

**(A)UMAP visualization of malignant cells from pontine and thalamic DMGs.**

**(B)Identification of region-specific marker genes in pontine and thalamic DMG tumor cells.**

**(C)GO enrichment analysis comparing pontine and thalamic DMGs.**

**(D)Metabolic pathway differences between pontine and thalamic DMGs, compared to other pHGG.**

**(E-G)SCENT assessed stemness of DMG and region-specific tumor cells.**

## Supplementary Figure 3


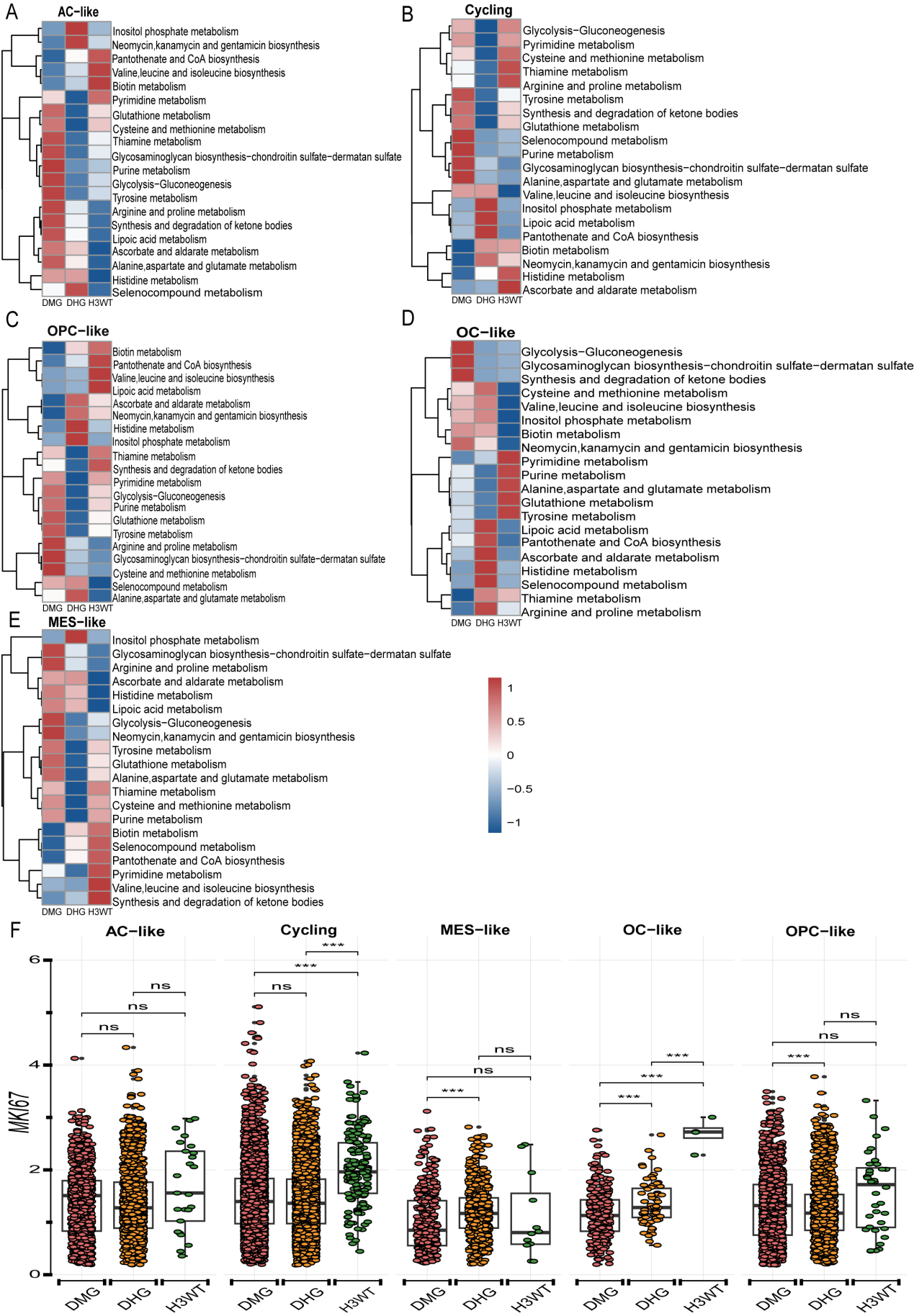


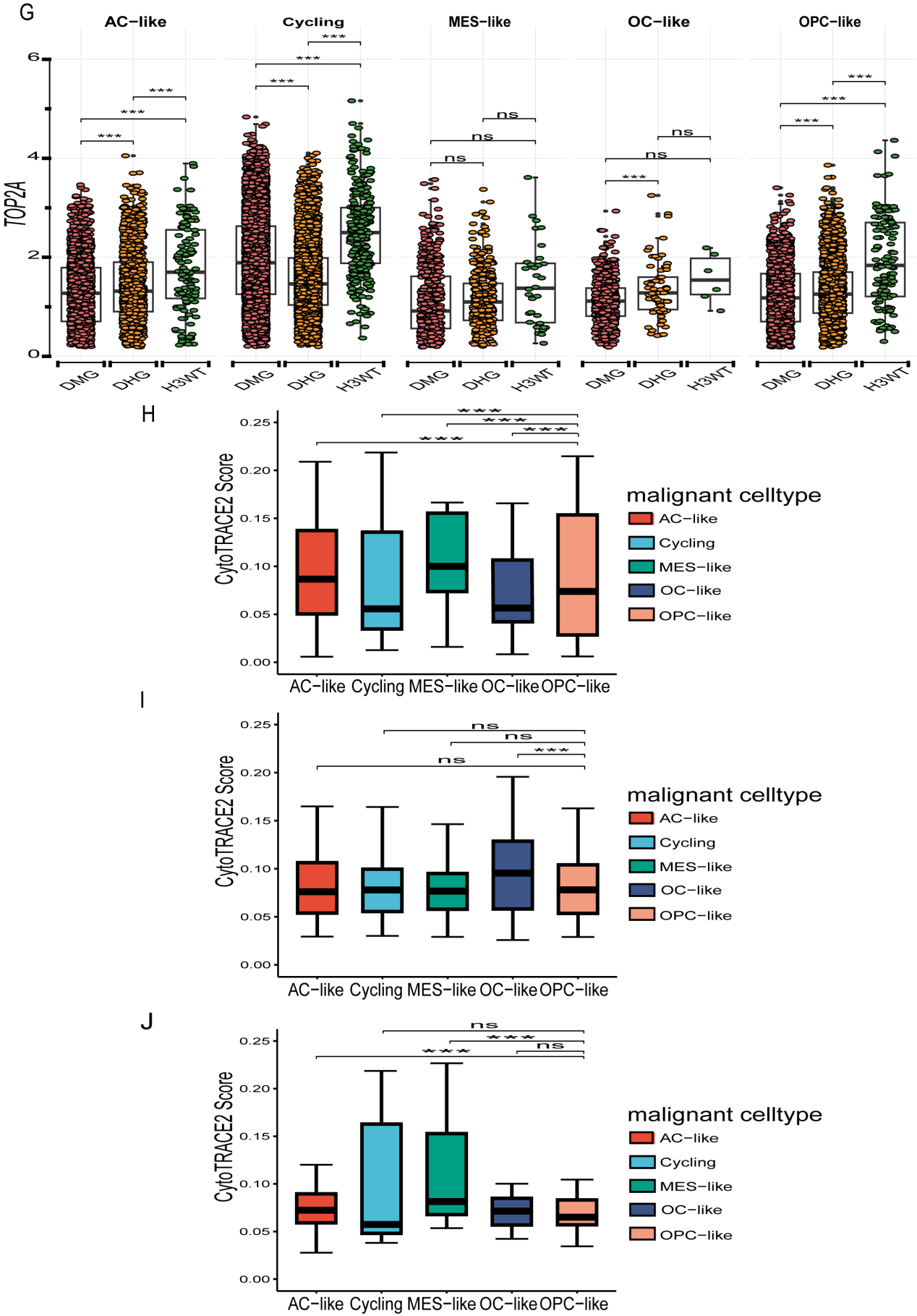


****Supplementary Figure 3: Characteristics and heterogeneity of malignant cells in pHGG.**(A–E) scMetabolism analysis of metabolic features across malignant cell subpopulations.
(F–G) Expression patterns of proliferation-related genes, MKI67 and TOP2A, in malignant cell subpopulations across different pHGG subtypes.**

## Supplementary Figure 4


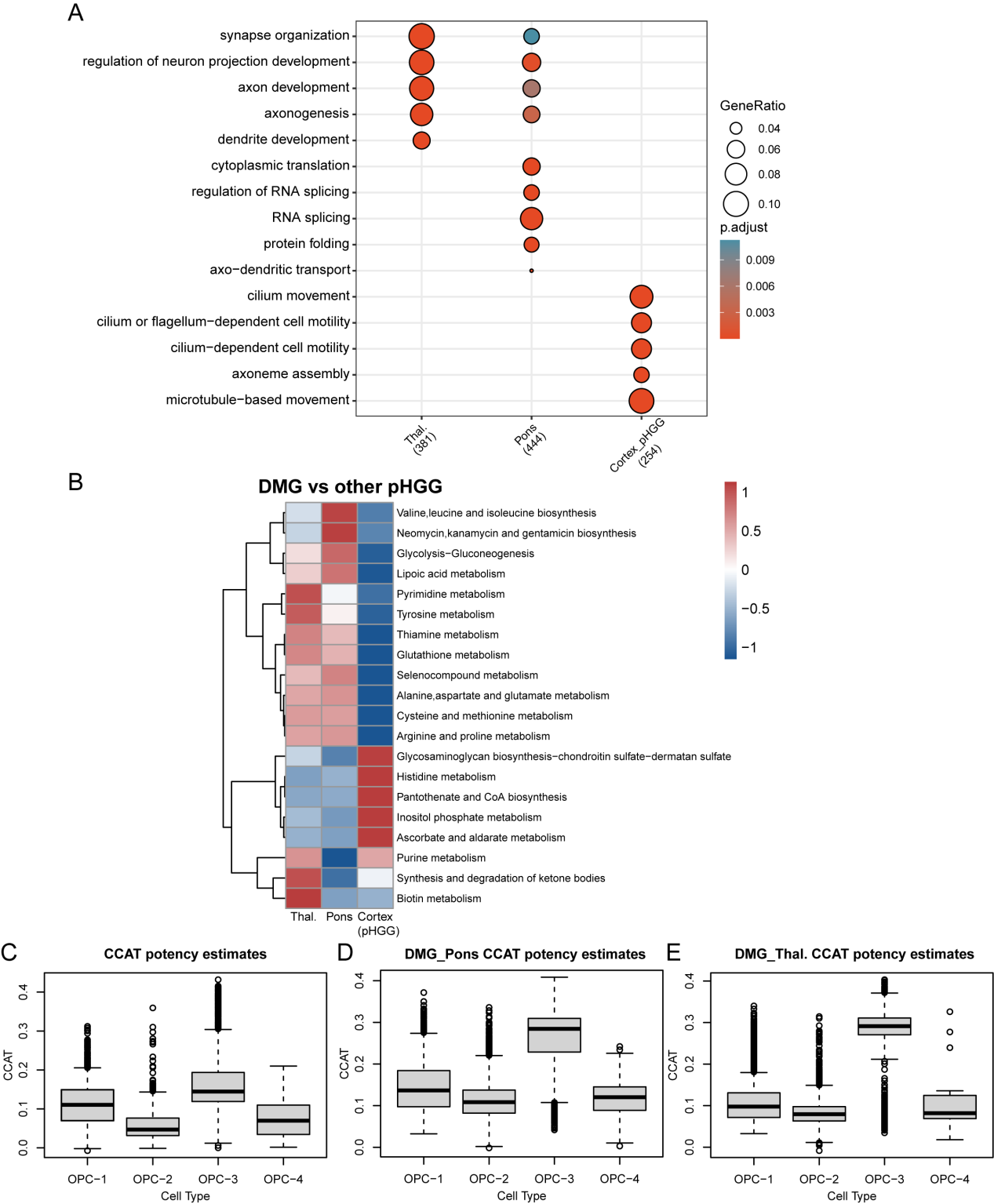


****Supplementary Figure 4: Biological characterization of region-specific OPC-like subpopulations in DMGs.****

1. **GO enrichment analysis comparing pontine and thalamic DMGs.**
2. **Metabolic pathway differences between OPC-like of pontine and thalamic DMGs, compared to other pHGG.**

**(C-E)SCENT assessed stemness of DMG and region-specific OPC-like.**
